# Advances in Iron-Based Superconductors and Transformational Insights into Electron–Differential Phonon Coupling

**DOI:** 10.3390/ma19061105

**Published:** 2026-03-12

**Authors:** Wai Kwan Liu, Ka Chun Li, Yanling Zhang, Chi Ho Wong

**Affiliations:** 1 Laboratory of Materials for Renewable Energy (LMER), Institute of Chemical Sciences and Engineering (ISIC), Basic Science Faculty (SB), École Polytechnique Fédérale de Lausanne (EPFL), CH-1950 Sion, Switzerland; wk.liu@epfl.ch; 2 EMPA Materials Science and Technology, CH-8600 Dubendorf, Switzerland; 3 Department of Chemical and Biological Engineering, The Hong Kong University of Science and Technology, Hong Kong, China; kcliad@connect.ust.hk; 4 Division of Science, Engineering, and Health Studies, The School of Professional Education and Executive Development, The Hong Kong Polytechnic University, Hong Kong, China; irene.zhang@cpce-polyu.edu.hk

**Keywords:** iron-based superconductors, electron-differential phonon couplings, *T_c_* calculations

## Abstract

Since the discovery of iron-based superconductors nearly two decades ago, significant advancements have been made, including the enhancement of the superconducting transition temperature ($T_{c}$) to above 100 K. However, the underlying pairing mechanism remains an unresolved enigma. In this article, we present experimental developments in iron-based superconductors, focusing on their unique properties and the complexities involved in their behavior. We discuss the recently announced electron–differential phonon coupling model, which aims to provide a framework to calculate the *T_c_* of iron-based superconductors, but raises questions about its applicability to all iron-based superconductors. We selectively analyze several compounds within the major iron-based families to assess their compatibility with the electron–differential phonon coupling model. By comparing experimental data with theoretical predictions, we identify which superconductors align with the model and which do not. Furthermore, our findings reveal several key reasons behind the discrepancies in calculating $T_{c}$ for those iron-based materials that fall outside the theoretical expectations. Despite this, the pairing mechanism of iron-based superconductors remains an open question.

## 1. Iron-Based High-$T_{c}$ Superconductors

We selectively survey the major experimental developments in iron-based superconductors and discuss how exotic quantum effects, as selectively observed in these systems, can be incorporated into an electron–differential phonon framework to bring the predicted *T_c_* closer to experimental values. We present both spin-fluctuation–mediated superconductivity and electron–phonon-based mechanisms, clarifying that we do not reject spin-mediated pairing; rather, we explore a potentially viable pathway in which exotic quantum effects couple to the electron–differential phonon channel to enhance pairing, where articulating a coherent framework that connects spin-strengthened pairing with phonon-carried pathways is possible.

### 1.1. History and Timeline of Iron-Based High-$T_{c}$ Superconductors

The history of iron-based superconductors begins with the pioneering work of Hideo Hosono and his collaborators, who fundamentally altered the prevailing understanding of superconductivity. Hosono’s group reported that the layered iron phosphide compound LaFePO exhibited superconductivity with a critical temperature *T_c_* of about 4 K in 2006 [1]. Although the transition temperature was low, the discovery was surprising because iron is intrinsically magnetic, and magnetic elements were long believed to be detrimental to superconductivity. This result suggested that layered iron pnictides could host unconventional pairing mechanisms, an idea that was only beginning to emerge at the time [2].

A decisive breakthrough occurred in 2008 with the discovery of superconductivity in LaOFeAs through electron doping. By partially substituting fluorine for oxygen, Kamihara, Watanabe, Hirano, and Hosono demonstrated that $La(O_{1-x}F_{x})FeAs$ exhibits a superconducting transition temperature of 26 K [3]. This landmark result overturned the long-standing assumption that iron-containing materials could not support high-*T_c_* superconductivity and immediately sparked worldwide interest. It marked the birth of iron pnictides as a new class of high temperature superconductors, distinct from both conventional phonon-mediated systems and cuprates.

Following this discovery, rapid progress was made through systematic chemical substitutions and structural tuning. Researchers found that replacing lanthanum with other rare-earth elements in the 1111-type REOFeAs family led to dramatic increases in $T_{c}$ [4,5,6]. Within a short period, superconductivity above 50 K was achieved in fluorine-doped and oxygen-deficient compounds, with optimized systems such as $SmO_{1-x}F_{x}FeAs$ reaching transition temperatures as high as 55 K [7]. These values remain the highest bulk critical temperature reported for iron-based superconductors to date.

From a timeline perspective, the field then expanded rapidly beyond the 1111 family. From 2008 to 2009, attention turned to structurally simpler 122-type ($A{Fe}_{2}{As}_{2}$) systems, which allowed easier crystal growth and extensive studies of doping- and pressure-induced superconductivity [8]. Almost concurrently, the 111-type (LiFeAs, NaFeAs) and 11-type (FeSe) compounds were identified, providing cleaner platforms with fewer atomic layers and enabling detailed investigations of electronic structure, magnetism, and pairing symmetry.

Together, these milestones established iron-based superconductors as one of the most important platforms for studying unconventional superconductivity, setting the stage for ongoing efforts to unify magnetism, lattice effects, and electronic correlations within a single theoretical framework.

Building on this chronological evolution, the discovery of superconductivity in the structurally simplest 11-type compound FeSe marked a pivotal shift in focus from chemical complexity to electronic reconstruction and interfacial effects. Unlike the earlier pnictides, FeSe exhibits strong electronic correlations, nematic order, and pronounced deviations from conventional Fermi-liquid behavior despite its relatively low bulk superconducting transition temperature. These characteristics made FeSe an ideal platform for exploring how magnetism, orbital physics, and lattice interactions cooperate to enhance superconductivity. This line of inquiry reached a climax with the realization that interfacing a single FeSe layer with $SrTiO_{3}$ can dramatically amplify the pairing interaction, producing superconducting energy scales and electron–phonon coupling far exceeding those of bulk materials. As such, FeSe and FeSe/STO form a natural bridge between the early discoveries in iron pnictides and the modern pursuit of interface-engineered high-*T_c_* superconductivity, motivating the unified theoretical framework discussed in the following sections.

### 1.2. Importance of Achieving High-$T_{c}$ in Iron-Based Superconductors

The discovery of high-*T_c_* superconductivity in iron pnictides was significant not only because of the surprise increase in transition temperature under anti-ferromagnetism (AFM), but also because of its far-reaching implications for materials science, condensed-matter physics, and technological applications [9]. From a practical standpoint, a higher superconducting transition temperature directly reduces reliance on liquid helium cooling, which is expensive and increasingly scarce. Traditional low-temperature superconductors require cooling to approximately 4 K, where the highest *T_c_* reported in bulk iron-based superconductors exhibit transition temperatures exceeding 55 K. This places them within reach of liquid nitrogen cooling (77 K), which is substantially cheaper, safer, and more scalable. As a result, iron-based superconductors are considered promising candidates for applications in power transmission cables, high-field magnets, fault-current limiters, and medical imaging systems such as magnetic resonance imaging (MRI), where operational cost and cooling simplicity are critical factors.

Beyond technological considerations, the unexpected high-*T_c_* superconductivity in iron-containing compounds profoundly altered the conceptual understanding of superconductivity itself. Prior to this discovery, magnetism, particularly from elements such as iron, was widely viewed as antagonistic to superconductivity, as magnetic moments tend to break Cooper pairs in conventional superconductors. The iron pnictides demonstrated that this assumption is overly simplistic. Instead, these materials revealed that magnetic interactions, especially antiferromagnetic spin fluctuations, can actively contribute to electron pairing rather than suppress it [10,11,12]. This realization shifted the theoretical paradigm by establishing spin-fluctuation–mediated pairing in multiband metals as a viable route to high-*T_c_* superconductivity, stimulating extensive theoretical and experimental efforts to understand unconventional pairing symmetries such as $s_{\pm }$.

More broadly, the success of iron-based superconductors revitalized the long-standing quest for room-temperature superconductivity by demonstrating that high-*T_c_* behavior is not exclusive to cuprates. Their remarkable tunability through chemical substitution, carrier doping, applied pressure, and structural or interfacial engineering provides an exceptional platform for systematically exploring how electronic correlations, lattice geometry, orbital degrees of freedom, and magnetism cooperate to enhance superconductivity. The subsequent discovery of 100 K superconductivity in systems such as monolayer FeSe on STO further underscored the importance of interface and lattice effects, opening new design strategies for achieving even higher transition temperatures. Together, these advances established iron-based superconductors as a cornerstone of modern superconductivity research and continue to guide the search for new materials with ever higher $T_{c}$.

### 1.3. Comparison Between Iron-Based and Conventional Superconductors

A fundamental distinction between iron-based superconductors and conventional superconductors lies in the microscopic mechanism responsible for Cooper pairing. Conventional superconductors, such as elemental metals including Pb and Al, are well described by Bardeen–Cooper–Schrieffer (BCS) theory, in which superconductivity arises from an attractive interaction between electrons mediated by lattice vibrations (phonons) [13]. This interaction leads to the formation of Cooper pairs and an isotropic s-wave superconducting gap. The electronic structures of conventional superconductors are typically characterized by relatively simple Fermi surfaces, weak electronic correlations, and strong sensitivity to magnetic impurities, which readily suppress superconductivity. Because the pairing interaction is limited by phonon energy scales, the superconducting transition temperatures of conventional materials are generally below 20 K, with ${MgB}_{2}$ which reaches $T_{c}$ = 39 K, representing a notable but still likely a phonon-mediated exception [14]. These fundamental contrasts between conventional and iron-based superconductors are summarized in Table 1, which highlights differences in pairing mechanisms, electronic structure, and superconducting properties.

Iron-based superconductors, by contrast, belong to the broader class of unconventional superconductors, where electronic correlations and anti-ferromagnetism play a central role in the pairing mechanism. Their parent compounds are typically antiferromagnetic metals, rather than the Mott insulators characteristic of cuprate superconductors. The electronic structure of iron pnictides and chalcogenides consists of multiple hole and electron Fermi-surface pockets, leading to intrinsically multiband superconductivity that cannot be adequately captured within a simple single-band BCS framework [15]. The close proximity of superconductivity and antiferromagnetism in these materials strongly suggests that antiferromagnetic interactions are intimately connected to the pairing process.

A widely accepted theoretical picture proposes that superconductivity in iron-based materials is mediated by spin fluctuations, giving rise to a sign-changing $s_{\pm }$ pairing symmetry [10,12,16,17]. In this scenario, the superconducting order parameter has opposite signs on the hole and electron pockets of the Fermi surface, a natural consequence of inter-band interactions enhanced by antiferromagnetic correlations. Depending on the details of the band structure and interaction strengths, the superconducting gap may be isotropic or anisotropic and may even host accidental nodes. Alternative pairing symmetries, including d-wave states, have also been discussed in regimes where the Fermi-surface topology is strongly modified [16]. J. Paglione et al. illustrate three representative possibilities for the superconducting order-parameter symmetry in iron-based superconductors [15]. The dominant theoretical picture suggests that the superconducting state arises from spin fluctuations, giving rise to a sign-changing $s_{\pm }$ pairing symmetry in which the superconducting gap has opposite signs on electron and hole pockets.

In addition to their unconventional pairing mechanism, iron-based superconductors exhibit remarkable structural flexibility. Small changes in structural parameters, such as the pnictogen height above the Fe plane, Fe-As bond angles, or lattice distortions induced by chemical substitution or applied pressure, can substantially modify the superconducting transition temperature and gap structure [18,19]. This pronounced tunability provides a powerful platform for exploring the interplay between lattice geometry, magnetism, and superconductivity, offering insights that conventional superconductors with their comparatively rigid structures cannot readily provide.

**Table 1:** Comparison between conventional and iron-based superconductors [7,12,14,17].

Feature	Conventional Superconductors	Iron-Based Superconductors
Representative materials	Hg, Pb, Al, Nb	FeSe, LaFeAsO,$\;Ba{Fe}_{2}{As}_{2}$
Pairing mechanism	Electron–phonon coupling (BCS)	Spin-fluctuation–mediated (unconventional)
Gap symmetry	Isotropic s-wave	Sign-changing $s_{\pm }$, anisotropic, or nodal
Electronic correlations	Weak	Moderate to strong
Parent state	Simple metal-based	Antiferromagnetic metal
Sensitivity to magnetism	Strongly suppresses SC	Magnetism often promotes pairing
Typical $T_{c}$	<20 K	Up to ~55 K (bulk), ~100 K (interfaces)

### 1.4. New Concepts and Technologies Enabled by Iron-Based Superconductors

The emergence of iron-based superconductors catalyzed several profound conceptual and technological advances that continue to shape modern superconductivity research. One of the most transformative insights was the realization that multiband superconductivity can support high transition temperatures through mechanisms fundamentally distinct from conventional electron–phonon coupling. In iron-based materials, the coexistence of complex Fermi-surface pockets enables pairing channels driven by electronic correlations and spin fluctuations, rather than purely electron–phonon interactions [10,17]. This recognition helped unify theoretical perspectives across seemingly disparate classes of unconventional superconductors, including cuprates, heavy-fermion systems, and iron pnictides, by emphasizing the central role of magnetically mediated pairing interactions in correlated electron systems.

Equally important was the demonstration that structural chemistry provides a powerful control parameter for superconductivity. Studies of iron-based superconductors revealed that subtle variations in structural motifs, such as the As-Fe-As bond angle, pnictogen height, layer stacking, applied pressure, chemical substitution, and interface engineering, can dramatically modify the superconducting transition temperature and gap structure [15,18,19]. This sensitivity established a new paradigm in which superconductivity could be systematically optimized through lattice and electronic structure engineering, moving the field beyond reliance on serendipitous discovery toward a more rational, design-driven approach to materials development.

Overall, iron-based superconductors have significantly expanded the conceptual landscape of superconductivity by clarifying the role of unconventional pairing mechanisms, demonstrating the critical importance of structural and interfacial engineering, and enabling systematic exploration of high *T_c_* behavior in correlated electron systems. Beyond their intrinsic scientific value, these materials have opened new pathways toward the development of next-generation superconducting technologies and theoretical frameworks. As such, the discovery of iron-based superconductors stands as one of the most important milestones in condensed-matter physics since the advent of high-*T_c_* cuprate superconductors.

## 2. FeSe

### 2.1. Introduction to FeSe: Simple Structure and Evolving $T_{c}$

FeSe is among the simplest members of the iron-based superconductor family because its crystal structure consists essentially of repeating FeSe layers [15]. In FeSe, a single iron layer is sandwiched between two selenium layers. This makes the FeSe layer the fundamental building block responsible for superconductivity in these materials. In its bulk form at ambient pressure, FeSe becomes superconducting at a relatively modest $T_{c}$ of about 8 K. However, $T_{c}$ can increase dramatically to 37 K under high pressure, illustrating the sensitivity of superconductivity to the structural and electronic environment [20]. More strikingly, in monolayer FeSe films grown on substrates such as $SrTiO_{3}$, experimental studies have reported much higher $T_{c}$ with some reports suggesting superconductivity possibly above 65 K [21,22]. Thus, FeSe represents a particularly appealing system: structurally simple, yet tunable over a wide range of superconducting transition temperatures by pressure, intercalation, or reduced dimensionality.

### 2.2. Differences in Electronic Distribution and Fermi-Level Features vs. Conventional Superconductors

In conventional superconductors, the low-energy electronic behavior is typically described by a relatively simple Fermi surface, often involving a single or a few conduction bands, where electrons near the Fermi level form Cooper pairs via electron–phonon interactions [23,24,25]. By contrast, in FeSe and related iron-based superconductors, the electronic structure is markedly more complex and fundamentally different [26]. In Fe-based systems, the low-energy states are dominated by the five Fe 3d orbitals. As a result, multiple bands cross the Fermi level, giving rise to multiple Fermi surface sheets, including hole-like pockets near the Brillouin zone center and electron-like pockets near the zone corners [27,28,29]. This multiband nature contrasts sharply with conventional simple-metal superconductors.

**Figure 1:**
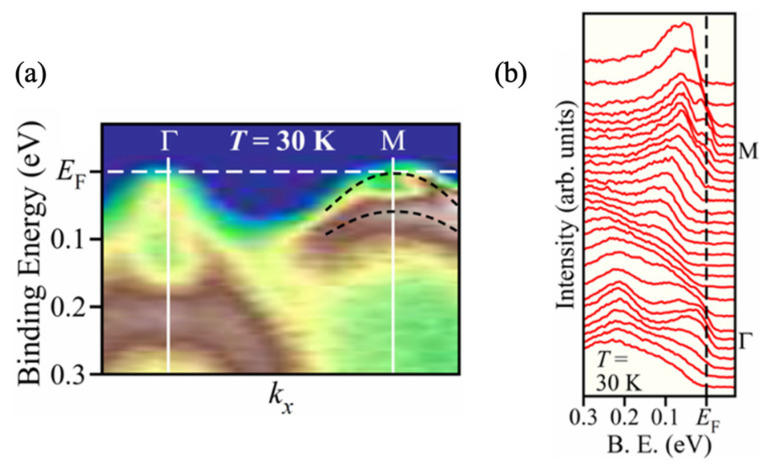
(**a**) ARPES intensity mapping and (**b**) corresponding EDCs for bulk FeSe along the Γ−M region at T = 30 K. The black dashed lines in (**a**) refer to the M-centered hole-like bands [30].

Moreover, angle-resolved photoemission spectroscopy (ARPES) experiments on FeSe single crystals reveal that the actual band dispersions and Fermi energies deviate significantly from typical band-structure: there is strong orbital-dependent renormalization, meaning electron correlations reshape the band structure in a way not captured by simple and weakly interacting electron gas models. Crucially, the experimental data show that the unusual electronic state below the Fermi level (abbreviation: the ARPES range) is responsible for the unconventional superconductivity in bulk FeSe [30]. This is comparable in magnitude to the superconducting gap and even, under certain conditions, to magnetic energy scales. As illustrated in Figure 1a, the band structure undergoes substantial reconstruction. These deviations provide strong evidence that the electronic distribution departs from ideal Fermi–Dirac statistics, supported by Figure 1b.

### 2.3. Implications: Fermi–Dirac Statistics, Quasiparticles, and Unconventional Superconductivity

The standard Fermi liquid or BCS picture assumes a well-defined Fermi surface. However, in most iron-based superconductors such as FeSe, the comparable energy scales between the Fermi energy, superconducting gap, and other energy scales, such as Zeeman energy, imply that a substantial fraction of electrons near the Fermi level may not behave like conventional quasiparticles [20]. Their distribution and pairing are strongly influenced by correlation effects between magnetism, electron–phonon coupling, etc., leading to non-trivial deviations from the ideal Fermi–Dirac distribution underlying conventional superconductivity. Thus, FeSe and related iron-chalcogenide superconductors provide a unique opportunity to explore physics in the iron-based regime. It may involve more localized or correlated electrons rather than delocalized electron states around a large Fermi surface [20].

Furthermore, the multiband nature in FeSe means that superconducting pairing likely involves inter-band scattering and complex orbital makeup, turning into non-s-wave pairing [31]. The variety of Fermi pockets and orbital contributions, including different Fe 3d orbitals open a rich phase space for unconventional pairing symmetries, anisotropic gaps, and possibly even exotic phases tied to electron correlation and orbital degree of freedom. This complexity suggests that the electron distribution at low temperature in FeSe is far removed from the simple, isotropic Fermi–Dirac sea of a conventional metal. It helps explain the unconventional superconducting behavior observed, including high $T_{c}$ in thin films, strong sensitivity to pressure, doping, and layer thickness.

## 3. FeSe/STO

### 3.1. Structural and Electronic Reconstruction in Monolayered FeSe/STO

**Figure 2:**
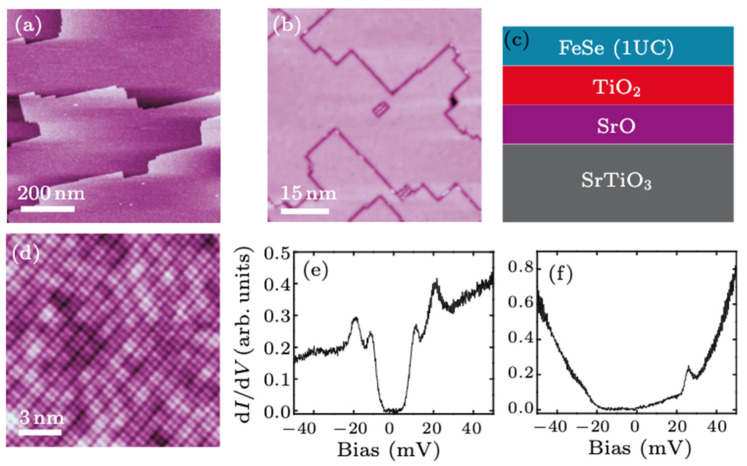
(**a**) STM image of annealed STO (001). (**b**) STM image of 1-UC FeSe/STO showing grain-boundary trenches. (**c**) Side-view schematic of the FeSe/STO interface. (**d**) Atomic-resolution STM image of the Se-terminated FeSe (001) lattice. (**e**) 1-UC FeSe/STO tunneling spectrum at 4.2 K showing a superconducting gap. (**f**) 2-UC FeSe spectrum displaying semiconducting, non-superconducting behavior [32].

FeSe is structurally the simplest member of the iron-based superconductor family, consisting of stacked FeSe layers where each iron atom is coordinated tetrahedrally by selenium. As shown in Figure 2a,b, STM topography reveals a clear surface after deposition of an approximately one-unit-cell (1 UC) thick FeSe film [32], where the schematic diagram is shown in Figure 2c. A higher-resolution STM image (Figure 2d) shows a well-ordered, Se-terminated (001) lattice [32]. Bulk FeSe possesses a modest superconducting transition temperature of roughly 8 to 9 K, but its electronic structure undergoes a profound transformation when reduced to a monolayer and grown epitaxially on $SrTiO_{3}$ (001) substrates [33,34]. In the monolayer limit, FeSe experiences significant strain, charge transfer, and interaction with substrate phonons, leading to a qualitatively different low-energy electronic landscape. ARPES measurements have shown that nematicity is involved in the pairing, resulting in an anisotropic Fermi surface [30]. Nematicity refers to a state in which the electronic system of a material breaks the rotational symmetry of its underlying lattice, reducing its apparent symmetry from fourfold (C4) to twofold (C2), while preserving its translational symmetry. In the context of iron-based superconductors, nematicity manifests as an in-plane electronic anisotropy that survives above the superconducting transition temperature and often precedes or accompanies other ordering phenomena. This electronic nematic order can lead to direction-dependent behavior in a range of physical properties, even when the crystal lattice remains tetragonal on average. Meanwhile, the atomic uniformity of the FeSe/STO and the presence of a large superconducting gap of 20.1 meV, shown in Figure 2e, are absent in the bulk material [32]. Together, these structural and electronic modifications establish the foundation upon which the enhanced superconducting properties emerge [32,35]. Figure 2f shows the contrasts in the non-superconductive FeSe film.

### 3.2. Interfacial Mechanisms Responsible for High-$T_{c}$ Enhancement

The dramatic enhancement of superconductivity in 1-UC FeSe/STO arises from a synergistic combination of interfacial effects, the most prominent of which is cross-interface electron–phonon coupling. $SrTiO_{3}$ hosts high-energy phonons associated with its ${TiO}_{2}$ surface layer. These phonon modes can couple strongly to electrons in the FeSe film, particularly through highly forward-focused scattering processes. ARPES experiments have revealed the direct spectral fingerprints of this strong interfacial electron–phonon coupling [36,37]. Theoretical models suggest that such forward-scattering phonons can significantly amplify the superconducting pairing interaction while avoiding the detrimental mass renormalization typically associated with phonon-mediated mechanisms [38]. Charge transfer from oxygen vacancies in STO, combined with substrate-induced strain and enhanced dielectric screening, further boosts pairing strength by enhancing the density of states and modifying orbital occupation [39]. Thus, the FeSe/STO interface represents a unique environment in which electronic correlations, phonons, and interfacial charge dynamics cooperate to raise the pairing scale far beyond that seen in freestanding or bulk FeSe [40].

### 3.3. Experimental Evidence for Superconductivity Approaching 100 K

The claim that monolayer FeSe on STO may achieve superconducting transition temperatures approaching 100 K originates from a series of landmark scanning tunneling spectroscopy and ARPES experiments. In these measurements, the superconducting gap magnitude in 1-UC FeSe/STO was found to reach values of 15 to 20 meV, nearly an order of magnitude larger than the bulk FeSe gap [32]. The temperature dependence of this gap, together with the persistence of coherence peaks at elevated temperatures, suggests that superconductivity may survive up to 65 to 80 K in many samples, and in exceptionally optimized films, possibly up to ~100 K [41]. The work published in Nature (2014) by Ge et al. reported the in situ four-probe electrical transport measurements of the 1-UC FeSe/STO heterostructure, indicating a superconducting transition point near 100 K [41].

While transport-based measurements often yield lower $T_{c}$ due to film inhomogeneity and phase-fluctuation effects, spectroscopic probes consistently show a pairing strength far exceeding that of any known Fe-based superconductor in bulk form. The presence of large, U-shaped gaps, complex band structures, etc., all support the existence of a robust superconducting state in the monolayer regime. Together, these findings establish FeSe/STO as the highest $T_{c}$ iron-based superconductor known to date and one of the most compelling examples of interface-engineered high-temperature superconductivity. The electron–differential phonon coupling model introduced by Chi Ho Wong and Rolf Lortz proposes that the variations in the critical temperature *T_c_* observed in FeSe/STO may stem from inconsistencies in interfacial strain [42]. Specifically, the results presented in Table 2 reveal that even a minor strain can significantly alter $T_{c}$.

## 4. FeAs

### 4.1. FeAs-Based Systems

The “FeAs system” refers broadly to a family of layered compounds in which superconductivity arises from Fe-As structural units [43]. The parent materials are typically poor metals (or semimetals) rather than insulators, and superconductivity is usually achieved by chemical doping, pressure, or structural tuning [44].

The understanding of Fe-based superconductors can be advanced from the simplest structural units to increasingly complex layered architectures. At the foundation lies the 11-type structure, exemplified by FeSe, which contains only Fe-Ch (chalcogen) layers without additional spacer blocks. The 11-type compounds illustrate how subtle tuning directly alters the Fe square lattice and induces superconductivity through doping, pressure, or structural modification.

Building on this simplest framework, introducing alkali-metal layers (Group 1 elements) between FeAs planes gives rise to the 111-type compounds. Prototypical examples include LiFeAs and NaFeAs, with Fe-As layers alternating with monovalent ion layers. LiFeAs is superconducting in its stoichiometric form, achieving a $T_{c}$ of ~18 K without any external doping or pressure [45]. Similarly, NaFeAs shows a $T_{c}$ of 10 K at ambient pressure [46]. Tuning the superconducting transition temperature of FeSe, LiFeAs, and NaFeAs through applied pressure has been shown to be effective. This approach effectively suppresses their antiferromagnetic ground state and amends superconductivity. The mechanisms behind how pressure influences these properties warrant further attention and investigation. However, not all Fe-based superconductors exhibit superconductivity at ambient pressure.

### 4.2. Pressure-Induced Fe-Based Superconductivity

Replacing the monovalent layers with divalent alkaline-earth ions (Group 2 elements) yields the 122-type compounds, ${AFe}_{2}{As}_{2}$ (A = Ba, Sr, Ca). These materials crystallize in the space group of I4/mmm, with FeAs layers separated by Ba/Sr/Ca layers [47]. The parent 122 compounds also exhibit antiferromagnetic order, but pressure readily induces superconductivity. When compared to the Sr and Ca cases, $Ba{Fe}_{2}{As}_{2}$ is a more extensively studied member of the 122-type iron pnictides due to high *T_c_*. Worldwide literature offers comprehensive studies on how hydrostatic pressure can induce superconductivity in this 122-type FeAs-based material. At ambient pressure, $Ba{Fe}_{2}{As}_{2}$ undergoes a coupled structural and antiferromagnetic transition above 100 K and remains non-superconducting, reflecting the strong stability of its magnetostructural ground state [48,49]. Applying pressure suppresses this magneto-structural order and drives the system into a superconducting state, highlighting the strong competition between antiferromagnetism and superconductivity that is a hallmark of iron pnictides [49,50].

**Figure 3:**
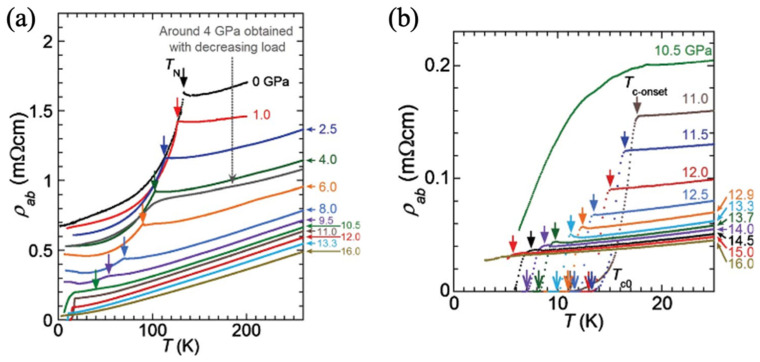
(**a**) Electrical resistivity of a $Ba{Fe}_{2}{As}_{2}$ single crystal as a function of temperature measured under different applied pressures using a cubic-anvil high-pressure technique. For comparison, resistivity data near 4 GPa collected during pressure release are also shown. (**b**) Enlarged low-temperature region of the resistivity curves at pressures exceeding 10 GPa. The superconducting transition onset temperature, $T_{c}^{onset}$, is identified from anomalies in the temperature derivative dρ/dT, while the zero-resistance transition temperature, $T_{c0}$, is defined as the point where the resistivity vanishes [51].

Figure 3 shows that superconductivity emerges in $Ba{Fe}_{2}{As}_{2}$ as a function of, with the superconducting transition temperature $T_{c}$ increasing rapidly as the magnetic order is suppressed [51]. The resulting pressure–temperature phase diagram exhibits a characteristic dome-shaped $T_{c}$ (*P*) dependence, with maximum $T_{c}$ values approaching 25 to 30 K, depending on experimental conditions and sample quality [51,52]. At higher pressures, $T_{c}$ decreases as the system moves away from the optimal balance between electronic correlations and lattice geometry. This pressure-induced superconducting dome closely parallels the behavior observed in chemically doped $Ba{Fe}_{2}{As}_{2}$, but without introducing chemical disorder [49].

Structural studies under pressure reveal that superconductivity in $Ba{Fe}_{2}{As}_{2}$ is strongly correlated with changes in key lattice parameters, including the Fe-As bond length, the arsenic height above the Fe plane, and the distortion of the FeAs tetrahedra. Structural modifications alter the electronic and magnetic exchange interactions, thereby highlighting the anisotropic feature of the Fermi surface and the strength of spin fluctuations that are believed to mediate the pairing [12,51,52]. Hence, pressure thus serves as an effective and continuous tuning parameter for triggering the intrinsic coupling between lattice, magnetism, and superconductivity in FeAs systems.

The pressure response of $Ba{Fe}_{2}{As}_{2}$ has also played an important role in the development of the theoretical models of iron-based superconductivity. Both first-principles calculations and semi-phenomenological approaches indicate that pressure simultaneously modifies magnetic interactions and electron–phonon coupling, reinforcing the view that superconductivity in FeAs materials arises from the cooperative interplay between magnetism and lattice degrees of freedom, rather than from a single dominant mechanism [12,16,51].

Beyond the overall magnitude of applied pressure, the anisotropy of the stress field has been shown to play a decisive role in $Ba{Fe}_{2}{As}_{2}$. Comparative high-pressure experiments indicate that superconductivity can be stabilized at lower effective pressures when a uniaxial stress component is present, even if the nominal pressure remains within the same range [51]. In particular, uniaxial compression along the crystallographic c axis can accelerate the suppression of the structural and spin-density-wave transitions, allowing superconductivity to emerge at pressures of approximately 1 to 2 GPa, compared with around 2 to 3 GPa under more hydrostatic conditions. This behavior reflects the strong sensitivity of magnetic order to lattice anisotropy in iron pnictides and highlights the importance of stress directionality in shaping the phase diagram. The role of uniaxial stress in shifting the balance between magnetism and superconductivity contrasts with nearly hydrostatic and anisotropic pressure conditions [52].

### 4.3. Doping-Induced Superconductivity

Doping introduces chemical pressure, which can also be used to trigger superconductivity in 122-type and 1111-type iron-based superconductors [53]. Their work on $Ba{Fe}_{2}{As}_{2}$ revealed that introducing hole carriers through K substitution effectively suppresses the stripe-type antiferromagnetic order of the parent phase and drives the material into a superconducting state. Interestingly, the authors also showed that external pressure produces a similar outcome, emphasizing that both chemical substitution and mechanical compression modify the Fe-As layers in ways that weaken the magnetic ground state and favor superconductivity. This parallel response highlights a unifying mechanism in which changes in electronic filling and lattice geometry combine to destabilize magnetism and promote pairing, further reinforcing the central role of carrier doping in inducing superconductivity across the FeAs family. With the help of dopants, $K_{x}{Ba}_{1-x}{Fe}_{2}{As}_{2}$ gives rise to the highest $T_{c}$ values at 38 K in the 122-family when x = 0.4 [48].

On the other hand, for 1111-type iron-based superconductors ($R_{E}$FeAsO, $R_{E}$ = rare earth) introduces additional chemical complexity in superconductivity [47]. These compounds adopt the space group of P4/nmm, where FeAs layers alternate with $R_{E}$-O blocks [54]. Crucially, charge carriers can be added by creating oxygen vacancies, enabling superconductivity in oxygen-deficient $R_{E}$FeAsO without extrinsic dopants. However, these oxygen-deficient phases often require high-pressure synthesis for stabilization.

The popular example of 1111-type iron-based superconductors is F-doped LaFeAsO. LaFeAsO does not exhibit superconductivity, even when pressure is applied. However, the transformation to a superconducting state can be achieved by doping with F atoms at an ambient pressure, resulting in the F-doped LaFeAsO superconductor [55], where it exhibits a high transition temperature of approximately 26 K at ambient pressure, with optimally doped samples achieving $T_{c}$ values of around 40 K at ~6 GPa. This observation highlights the influence of doping and pressure on enhancing superconductivity.

More specifically, when fluorine was substituted at the oxygen site in $LaFeAsO_{1-x}F_{x}$, which injects extra electrons into the Fe-As layers while simultaneously introducing some degree of disorder [3]. Because the ground state of undoped FeAs materials is a long-range antiferromagnet, which generally competes with superconductivity, electron doping likely suppresses this magnetic order and enables superconductivity to emerge. Replacing La with Sr introduces charge carriers into the Fe-As plane, although the resulting superconducting transition temperatures are noticeably lower [56,57]. Substituting La with other cations, such as Th or Pb, has also been shown to induce superconductivity [58,59]. The highest bulk *T_c_* values in the FeAs system are observed in the 1111-type SmFeAsO-based compounds, which can reach up to 55 K [7,43,60]. This connection suggests that further optimization in doping and structural modifications can drive the superconducting properties of FeAs-based materials to even higher transition temperatures.

Doping can also be performed directly within the Fe-As layer. Substituting Fe with Co or Ni introduces electron carriers and partially replacing As with P alters the electronic structure, though in all cases the resulting transition temperatures are lower compared to F- or oxygen-vacancy-doped compounds [61,62]. These results collectively demonstrate that all four atomic sites in the 1111 structure can be chemically tuned to induce superconductivity. Notably, the fact that superconductivity survives Co substitution directly on the Fe site highlights the robustness of the pairing state in iron-based superconductors, in stark contrast to cuprates, where such in-plane disorder is strongly detrimental. Apart from these, oxygen-free 1111-type compounds such as CaFeAsF and SrFeAsF have attracted attention. These materials, which replace the $R_{E}$-O layer with $A_{E}$-F ($A_{E}$ = alkaline-earth), also become superconducting upon Co doping or rare-earth substitution [63,64,65].

Across all Fe-based materials, ranging from the simplest 11-type to the more complex 111, 122, and 1111 types, the chemical and structural flexibility provided by doping and pressure allows for systematic tuning of their electronic structures. This tunability offers a strategic platform for exploring how lattice parameters, pnictogen height, carrier concentration, and interlayer coupling collectively govern high-$T_{c}$ superconductivity in the iron pnictides.

## 5. Magnetically Enhanced Electron–Phonon Coupling in Fe-Based Superconductors

Despite their relatively high critical temperatures, the conventional Bardeen-Cooper-Schrieffer (BCS) theory cannot account for superconductivity in Fe-based materials [66,67]. Standard BCS theory relies solely on electron–phonon coupling and predicts that the transition temperature scales with the strength of the electron–phonon matrix element g [13]. However, first-principles calculations show that the intrinsic electron–phonon interaction in Fe-based superconductors is far too weak to explain the experimentally observed $T_{c}$ values [10,68,69]. This discrepancy has prompted the early development of hybrid theories in which magnetism cooperatively enhances phonon-mediated pairing [66] because the main difference in observation is that conventional superconductors do not have a magnetic moment, while the Fe-based superconductors have. However, the antiferromagnetic (AFM) fluctuations can only enhance electron–phonon coupling by approximately 20–30%, which is still far below the experimentally observed superconducting transition temperature.

In iron-based superconductors, the sole electron–phonon coupling is unlikely to be the primary mechanism for superconductivity. Spin- and multiorbital–mediated pairing formula remains an open question, with experimental evidence suggesting that spin-density-wave (SDW), charge-density-wave (CDW) phenomena, and multiorbital interaction (exotic quantum effects) play a central role in the pairing mechanism. Building on this trend, Wong and Lortz propose to amplify Cooper-pair bonding by leveraging exotic spin and multiorbital interactions through an electron–differential phonon-mediated channel. In other words, the electron–phonon coupling would not be the dominant driver of *T_c_*, but would serve as the medium by which exotic quantum effects are transferred to the Cooper pairs. The new aspect of our approach is to incorporate these exotic quantum effects into the electron–phonon coupling framework, rather than attempting to infer *T_c_* from exotic mechanisms alone.

### 5.1. AFM Spin Density Wave as the Potential Booster of $T_{c}$

A crucial insight into the unconventional superconducting mechanism of iron-based materials was provided by Coh et al., who demonstrated that the effective strength of the electron–phonon interaction depends sensitively on the magnetic configuration of the Fe sublattice rather than on the mean-field values [66]. Using first-principles calculations that explicitly account for magnetic order, they showed that magnetic arrangements can fundamentally alter the symmetry properties of the electronic wavefunctions that enter the electron–phonon matrix element g.

**Figure 4:**
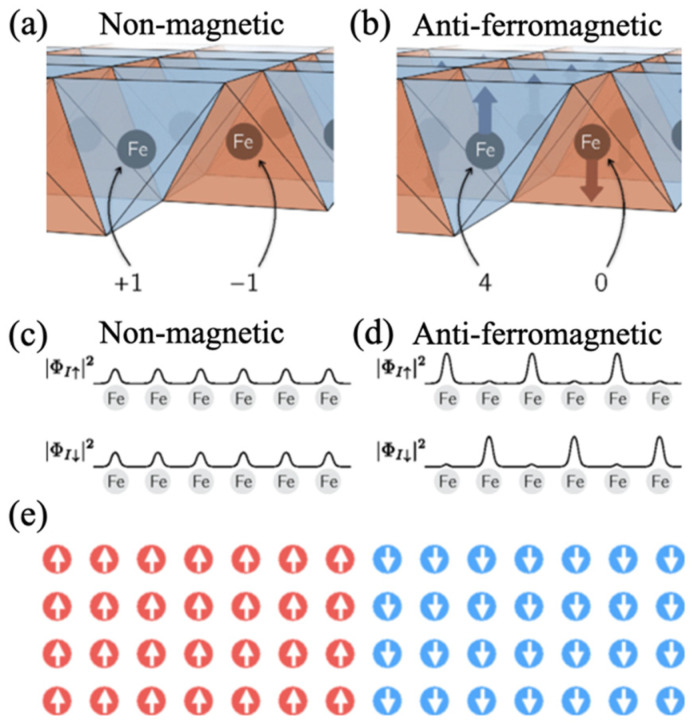
Electron–phonon matrix of two Fe atoms in (**a**) the non-magnetic state, and (**b**) the antiferromagnetic state. Electron orbital amplitudes in (**c**) the non-magnetic state, and (**d**) the antiferromagnetic state. (**e**) Globally antiferromagnetic yet locally ferromagnetic Fe spin arrangement (red/blue circles represent Fe atoms with opposite spin orientations) [66].

In the non-magnetic state, the primitive unit cell contains two crystallographically equivalent Fe atoms whose electronic states are related by symmetry. As illustrated schematically in Figure 4a, the lattice distortion associated with a given phonon mode induces contributions to the electron–phonon matrix element from each Fe site with opposite phases. These contributions therefore cancel almost exactly, resulting in an extremely small net matrix element, consistent with the low superconducting transition temperatures expected from conventional phonon-mediated pairing in iron-based compounds when magnetism is neglected [66,68].

In contrast, the situation changes qualitatively in the presence of an antiferromagnetic spin density wave. In the parent compounds of iron-based superconductors, the low-energy electronic ground state is characterized by a spin-density-wave (SDW) order, arising from Fermi-surface nesting between hole and electron pockets. This SDW state leads to a spatial modulation of the spin density on the Fe sublattice and is accompanied by a substantial reconstruction of the electronic structure, particularly involving the Fe 3d orbitals that dominate the states near the Fermi level [70]. As shown in Figure 4b, the electronic wavefunctions on the two Fe sites are no longer equivalent, and their respective contributions to the electron–phonon matrix element no longer cancel. Instead, each Fe atom contributes a finite component with the same sign, leading to a substantial two-fold enhancement of the local electron–phonon scattering matrix due to the conservation of antiferromagnetic energy from the minimal to the maximal spin sites [66].

This mechanism implies that antiferromagnetic spin density waves could enhance local electron–phonon coupling in a periodic manner. As a result, phonons that would be coupled strongly to itinerant electrons could become significantly more effective pairing mediators in an antiferromagnetically fluctuating background. This insight provides an explanation for how phonons may contribute constructively to superconductivity in iron-based materials without contradicting the widely observed importance of magnetic correlations [66,68].

The SDW order can be briefly described by the modulation of the local spin density. This magnetic modulation breaks the equivalence of Fe sites within the crystallographic unit cell, redistributing magnetic spectral weight and reinforcing the orbital-dependent electronic reconstruction [70].

As illustrated schematically in Figure 4a–d, the emergence of antiferromagnetic order periodically enhances the amplitude on specific Fe spin sites. The resulting spatial variation in the electronic wavefunctions increases the local overlap between electronic states and lattice vibrations. Microscopically, this effect enters through the electron–phonon matrix element:

$$g_{k,k+q}^{v}=\langle \psi_{k+q}\left|\frac{\partial V}{\partial u_{v}}\right|\psi_{k}\rangle ,$$

where $u_{v}$ denotes a phonon displacement, and V is the potential. In the presence of magnetic order, the reconstructed wavefunctions $\psi_{k}$ yield a significantly enhanced matrix element, particularly for phonon modes involving Fe–As bond stretching and Fe-plane distortions [71,72].

This enhancement is magnetically driven rather than phonon in origin. First-principles calculations show that antiferromagnetic order lifts the symmetry-induced cancellation of contributions from the two Fe atoms in the unit cell, resulting in a 4-fold increase in the effective electron–phonon coupling λ:

$$\lambda \propto \sum_{v,q}\frac{{\left|g_{v}(q)\right|}^{2}}{\omega_{v}(q)},$$

even without substantial phonon softening [73]. As a result, the spin density wave acts as a 4-fold booster of local electron–phonon coupling.

This cooperative mechanism satisfies the fundamental requirement of BCS-type pairing while overcoming the weak-coupling limitation of conventional electron–phonon superconductors. Spin density waves, therefore, do not simply compete with superconductivity in iron-based materials; instead, it reshapes the spin patterns in a way that allows phonons to contribute locally to pairing at the constructive spin sites, providing a promising pathway toward enhanced superconducting transition temperatures [73].

### 5.2. From Magnetic Modulation to Charge-Density Modulation

Beyond the spin degree of freedom, magnetic order in iron-based superconductors also imposes charge-density waves (CDW), as the change in magnetic flux across the high-spin and low-spin Fe sites induces an electric potential governed by Maxwell’s equations. Iron-based superconductors exhibit a rich spatial CDW structure due to the interplay of multi-orbital electronic states and antiferromagnetic order. In these systems, electrons near the Fermi surface are redistributed into regions of enhanced and suppressed charge density, giving rise to a charge-density modulation that is intertwined with magnetic and orbital degrees of freedom [74,75,76]. Although the long-range magnetic order remains antiferromagnetic, local regions emerge in which neighboring Fe moments are aligned ferromagnetically due to enhanced orbital amplitudes and locally increased charge density [74,75]. These ferromagnetic patches are not separate phases but are embedded within the antiferromagnetic background and are stabilized by the multiorbital nature of the Fe 3d electrons.

It has been noted that the induced electric potential across the differential magnetic flux between adjacent Fe atoms affects the electrostatic interaction of electrons. This induced electric potential could redistribute electronic charges in the form of charge density waves under an antiferromagnetic spin density wave background. This induced electric potential around the tetrahedral regions has been observed consistently, where Coh et al. calibrated the GGA + A functional to match the experimental findings. They discovered that the FeSe lattice, under the calibrated GGA + A functional, exhibits an induced *xy*-potential that further approximately doubles the electron–phonon scattering matrix.

The appearance of the induced potential is not surprising, as magnetism can slow down phonon vibrations. More specifically, the high-spin sites in antiferromagnetism reduce lattice vibrations compared to the low-spin sites. Consequently, the lattice vibrations between adjacent Fe atoms differ under the spin density wave pattern. These abnormal phonon (or differential phonon) behaviors across the magnetic boundaries trigger local electric polarization in the form of the induced potential. The change in lattice vibration along the out-of-plane direction is more pronounced because the tetrahedral atoms are pulling along the out-of-plane axis. This electric polarization induces charges in the *xy*-plane more effectively due to the long out-of-plane c-axis. This induced potential could continuously adjust the wavelength of the charge density wave rather than fix it to alternating lattice points, depending on the strength of the potential induction. These locally enhanced charge-density regions coincide with points of maximum electronic probability density or the electronic density of states on the reconstructed Fermi surface. At these locations, the overlap between electronic wavefunctions and lattice vibrations is significantly increased, leading to a higher probability of electron–phonon scattering. As a result, the charge-density modulation induced by the antiferromagnetic spin density wave not only reshapes the magnetic landscape but also creates local “hot spots” where phonon-mediated interactions are strongly amplified. This provides a periodic channel through which magnetic order can indirectly enhance superconducting pairing in iron-based materials [75,76].

### 5.3. Local “Hot Spots” for the Electron–Phonon Interaction

The presence of locally enhanced charges and spin density on selected Fe sites creates effective “hot spots” for electron–phonon interactions, where lattice vibrations couple unusually strongly to low-energy electronic states [66,73]. We refer to these intricate effects as electron–differential phonon coupling. At these sites, phonon modes involving Fe-As bond stretching and Fe-plane distortions experience a markedly increased overlap with reconstructed electronic orbitals. The electron–differential phonon coupling may lead to a significant amplification of $T_{c}$, even in the absence of large Debye temperatures. While superconductive pairing can be dominated by electronic states near the Fermi surface, these periodic “hot spots” may contribute to the pairing interaction. As a result, although the electron–phonon coupling remains spatially inhomogeneous, the alternating sites may inherit an effectively enhanced pairing strength, allowing the superconducting transition temperature to exceed values expected from conventional phonon-mediated mechanisms alone [66].

From a broader perspective, this behavior reflects a cooperative hierarchy of interactions unique to iron-based superconductors. Antiferromagnetic order, spin-density-wave, and charge density waves reconstruct the electronic structure on top of conventional electron–phonon coupling; these could trigger an anisotropic momentum space of electrons on the Fermi surface. These electronic reconstructions, in turn, create favorable conditions for differential phonons to couple more efficiently to electrons at specific momentum and real-space locations. The resulting magnetically assisted electron–phonon mechanism thus emerges from the intertwined nature of spin, charge, orbital, and lattice degrees of freedom [71,77].

This hybrid mechanism could provide insight into puzzling the theory of iron-based superconductors, including their ability to sustain high superconducting transition temperatures. By unifying phononic and magnetic contributions within this framework, it may offer a compelling pathway for understanding why iron-based superconductors lie well outside the expectations of conventional BCS theory while still retaining some key elements of phonon-assisted pairing [66,71,77].

## 6. Semi-Phenomenological Modeling Framework for Electron–Differential Phonon Interactions

Understanding superconductivity in iron-based materials requires a theoretical framework that goes beyond conventional electron–phonon coupling. Experimental evidence shows that there is a very large electron–phonon coupling in the monolayered FeSe/STO composite. As the simplest Fe-based superconductor, the FeSe monolayer highlights the importance of incorporating electron–phonon coupling into the theoretical framework. On top of the electron–phonon coupling, additional interactions reshaping the pairing mechanism should be considered. Recent advances demonstrate that magnetism, particularly antiferromagnetic (AFM), spin-density-wave (SDW), and charge-density-wave (CDW) correlations, can dramatically modify electronic states. Building on this insight, several studies have developed semi-phenomenological models that begin with density-functional theory (DFT)-based electron–differential phonon coupling that systematically reconsiders the magnetic, structural, and the unusual distribution of electrons below the Fermi level in the ARPES data [78,79,80,81]. This semi-phenomenological approach allows quantitative comparison across different iron-based superconductors, demonstrating how the computed $T_{c}$ values converge toward the experimental values even though the match is not perfect.

### 6.1. Role of the Orbital Degree of Freedom in Iron-Based Superconductors

C.H. Wong and R. Lortz developed a semi-phenomenological framework to simulate the phase diagrams and $T_{c}$ values of 11-, 111- and 122-type iron-based superconductors by starting from the concept of the electron–differential phonon coupling [77].

As electrons and phonons build up interactions, antiferromagnetic energy slows down the phonons, and maximum spin sites further slowdown phonon, which enhances electron–phonon coupling. Meanwhile, the differential spin sites across the spin density wave trigger the induction of electric charges or potential periodically. These induced charges, along with the original electrons, produce additional electrostatic interactions that not only modulate the wavelength of the charge density wave but also amend the local electronic density of states. This additional electrostatic interaction could lead to an anisotropic Fermi surface. In the presence of induced charges and the electrons within the ARPES range in the lattice, the dielectric screening effect changes, which simultaneously affects electron–phonon coupling. While some complex exotic effects (e.g., nematicity) may not be fully accounted for, these effects are primarily reported to influence the symmetry of the Fermi surface. If we capture the changes in the Fermi surface, we partially account for the effects of these complex phenomena.

The bare electron–phonon coupling $\lambda_{bare}$ is obtained in the usual Eliashberg form:

$$\lambda_{bare}=2\int_{0}^{\infty }\frac{\alpha^{2}F(\omega )}{\omega }d\omega$$

where $\alpha^{2}F(\omega )$ is the Eliashberg spectral function computed from CASTEP-based electronic and phonon calculations [77].

Since their model does not cover one-dimensional superconductivity, the electronic density of states around the Fermi level can be treated as constant. They then approximate several multiplicative linear separation-of-variable factors and define an effective pairing strength $\lambda_{eff}$ as follows:

$$\lambda_{eff}=\lambda_{bare}\cdot f_{ex}^{n}(P)\cdot R_{CDW}^{2}\cdot R_{ARPES}^{2}{\;\cdot \;f}_{angular}\cdot R_{SDW}^{2}$$

The second benefit of writing these expressions is that it allows for easier reproduction of the results, as others can observe how each factor changes individually. If all the factors are placed inside the Eliashberg integral, the $T_{c}$ values do not change significantly [78].

Each factor encodes a distinct piece of physics:

(1) Exchange factor $f_{ex}(P)$^n^

Encodes the pressure dependence of the mean-field antiferromagnetic exchange interaction, constructed from the Fe magnetic moment $M_{Fe}(P)$ and exchange-correlation energy $E_{co}(P)$. A simple normalized form is used, e.g.,

$$f_{ex}(P)\propto \frac{M_{Fe}(P)}{M_{Fe}(0)}\cdot \frac{E_{co}(P)}{E_{co}(0)}$$

so that $f_{ex}(0)\approx$ 1, referring to the case at ambient pressure. The order of antiferromagnetic fluctuations is n.

(2) Charge-density wave factor $R_{CDW}$ [66]

Motivated by Coh et al.’s result that the abnormal phonon can enhance the electron–phonon matrix element by about a factor of 2, Wong and Lortz parameterize this as CDW factor:

$$R_{CDW}\sim \;1.5-2.5$$

where the exact value of $R_{CDW}$ depends on the sample, which can be generally interpreted from the two-channel model [77] or through empirical calibration of DFT functions with experimental data [66].

(3) ARPES factor $R_{ARPES}$

To include electrons involved in the ARPES spectral-weight shift below $E_{F}$, the electron–phonon scattering matrix $g_{pp}(E)$ is averaged over an energy window $[E_{F}-\hbar \omega_{D},\;E_{F}$] and compared to its value at the Fermi level:

$$R_{ARPES}=\frac{{\langle g_{pp}(E)\rangle }_{E_{F}-\hbar \omega_{D}}^{E_{F}}}{g_{pp}(E_{F})}$$

where $g_{pp}(E)$ includes dielectric screening corrections (effective ε) appropriate to the ARPES energy range after the emergence of charge density waves.

(4) Gap anisotropy factor $f_{angular}$

An anisotropic gap ∆(θ) on the Fermi surface is modeled via an angular average:

$$f_{angular}=\frac{1}{2\pi }\int_{0}^{2\pi }\frac{\left|∆(\theta )\right|}{{∆}_{0}}d\theta$$

with ${∆}_{0}$ a reference gap scale. For an isotropic s-wave gap, $f_{angular}$ = 1; anisotropy reduces the effective coupling.

(5) Spin-density-wave factor $R_{SDW}$ [66]

When a spin density wave forms under an antiferromagnetic background, the spin energy at even sites transfers to odd sites, resulting in a minimal spin density at even sites and doubling the spin density at odd sites. The maximum spin density at odd sites amplifies the antiferromagnetically enhanced electron–phonon scattering by a factor of 2 due to the conservation of magnetic energy (or vice versa between odd and even sites). Therefore, and hence $R_{SDW}$ = 2.

The resulting $\lambda_{eff}$ is then fed into a McMillan-type expression [80] to estimate the superconducting critical temperature:

$$T_{C}=\frac{T_{D}}{1.45}exp\left[-\frac{1.04(1+\lambda_{eff})}{\lambda_{eff}-\mu^{*}(1+0.62\lambda_{eff})}\right]$$

where $T_{D}$ is a Debye (or characteristic phonon) temperature and $\mu^{*}$ is the Coulomb pseudopotential (typically $\mu^{*}\approx 0.15$) [79].

In other words, Spin Density Wave (SDW) factors arise from symmetrical considerations, or from the conservation of electron–phonon scattering probabilities. Charge Density Wave (CDW) factors are calculated by a two-channel model, where the two channels are integrated within a first-principles framework and calibrated against FeSe experiments. In angle-resolved photoemission spectroscopy (ARPES), the relevant energy range is set by the maximum phonon frequency, i.e., the Debye frequency. When a CDW emerges, the electronic distribution changes, which in turn alters the dielectric constant. This dielectric modification can be computed within first-principles calculations.

**Figure 5:**
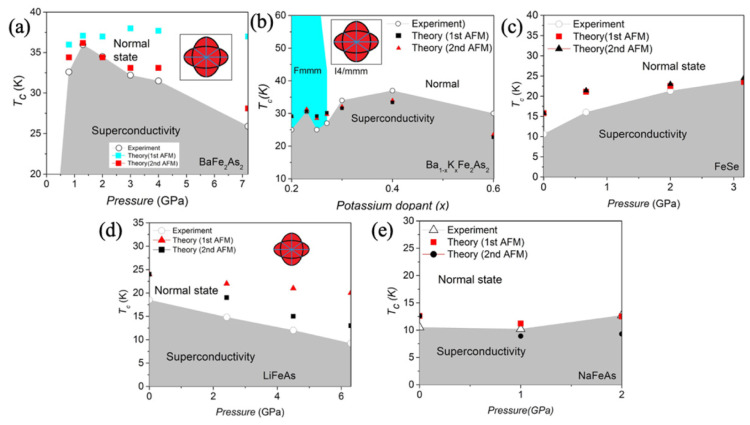
Pressure dependence of the experimental and theoretical ${\;T}_{c}$ in (**a**) $Ba{Fe}_{2}{As}_{2}$; (**b**) ${Ba}_{1-x}{K_{x}Fe}_{2}{As}_{2}$ with the change in ${\;T}_{c}$ along increasing potassium dopant; (**c**) FeSe; (**d**) LiFeAs; (**e**) NaFeAs [42,77].

Within this framework, as presented in Figure 5, Wong and Lortz reproduce the pressure- and doping-dependent $T_{C}$ of $Ba{Fe}_{2}{As}_{2}$, ${Ba}_{1-x}{K_{x}Fe}_{2}{As}_{2}$, $Li{Fe}_{2}{As}_{2}$, and $Na{Fe}_{2}{As}_{2}$, and reasonably predicts the absence of superconductivity in $Mg{Fe}_{2}{As}_{2}\;$[77].

### 6.2. Extension of the Framework to FeSe, FeSe/STO, LiFeAs, and NaFeAs

In a subsequent work focusing on FeSe and FeSe/STO, Wong and Lortz also introduced the concept of a synergistic energy $H_{synergy}$ to quantify how spin-density waves (SDW), charge-density waves (CDW), and differential phonons jointly reshape the electron distribution below the Fermi level [80]. Rather than focusing directly on dimensionless λ, they calculated an effective energy scale to the width of the ARPES redistribution window (~30–300 meV below $E_{F}$). 

For bulk FeSe, the computed synergistic energy is ~25–30 meV, consistent with the observed ARPES redistribution window in FeSe [80].

For monolayer FeSe/STO, the same structure is used, but $R_{SDW}$ and especially $R_{CDW}$ increase due to stronger interfacial effects and enhanced electron–phonon coupling at the FeSe/STO interface. The authors found that the computed synergistic energy is above 300 meV, matching the much larger ARPES energy range and consistent with the strongly enhanced superconductivity in FeSe/STO compared with bulk FeSe [80].

While this “synergistic energy” is not a conventional Eliashberg λ, it plays an analogous role as an effective pairing energy scale that integrates AFM, SDW, CDW, and anisotropy into a single quantity that can be compared across materials.

Although the synergistic-energy framework predicts a strong enhancement of superconductivity in monolayer FeSe/STO, experimental results show a remarkably wide spread of transition temperatures [41]. Table 2 reported ${\;T}_{c}$ values for FeSe/STO span from the idealized upper limit near 100 K observed only under highly optimized growth conditions to much lower values (19 K), and in some samples, superconductivity is entirely absent [42]. This variability underscores the extreme sensitivity of the interfacial superconducting state to substrate quality, oxygen vacancy concentration, and, critically, the surface *xy*-strain imposed by lattice mismatch between FeSe and STO. While monolayer FeSe/STO can in principle reach superconducting transitions near 100 K, most experimental realizations fall far short, demonstrating that precise interface engineering is essential for stabilizing the high-$T_{c}$ regime.

Moreover, experimental and theoretical studies reveal a clear hierarchy in how iron-based superconductors deviate from ideal Fermi–Dirac statistics, as illustrated in Figure 5. Among these materials, NaFeAs stands out as the closest to the ideal Fermi–Dirac statistics, exhibiting sharp quasiparticle peaks and a clean occupation profile that follows the expected thermal distribution [42]. LiFeAs shows modest departures of the Fermi–Dirac from ideality [42].

In contrast, FeSe/STO displays very profound non-Fermi–Dirac characteristics, most notably a substantial loss of spectral weight extending as deep as 300 meV below $E_{F}$ as observed by ARPES [42,80]. Such behavior cannot be explained simply by the BCS theory; instead, it reflects extensive electronic reconstruction driven by spin-density-wave tendencies, charge redistribution, orbital polarization, and interfacial phonon coupling. Importantly, when these effects are included in modern theoretical frameworks such as the synergistic-energy model, the resulting effective electronic energy scale naturally reproduces this ~300 meV window, demonstrating excellent consistency with experimental data from FeSe/STO [80].

Taken together, these findings highlight a unifying theme across the iron-based superconductors: while systems like NaFeAs lie close to the conventional Fermi-liquid limit, FeSe and its heterostructures operate in a profoundly reconstructed electronic regime where magnetism, charge order, and lattice interactions cooperate to reshape low-energy quasiparticles. This hierarchy of electronic coherence not only clarifies the diverse physical behavior among Fe-based families but also reinforces the broader modeling framework. It shows that superconductivity in these materials emerges from the delicate interplay between electron correlations, magnetic fluctuations, and lattice coupling. As such, these insights provide a natural conclusion to the unified perspective developed in this section.

### 6.3. The Incapability of the Electron–Differential Phonon Coupling Model in Some Regimes

Although the electron–differential phonon coupling model can calculate the $T_{c}$ of some iron-based superconductors (Table 3), it does not correspond well with 1111-type iron-based superconductors. This discrepancy is likely due to the neglect of electron doping within the tetrahedral region. Additionally, their two-channel model serves as a general approximation, while a more accurate approach would require time-consuming empirical calibration with experimental data. Moreover, modeling dielectric properties can be challenging due to the charge dynamics present under charge density wave conditions. The antiferromagnetic exchange factor under pressure can be approximated by mean field theory, which may not adequately address the complex spin and charge coupling in 1111-type superconductors. Wong and Lortz did not test their model on all reported 122, 11, and 111-type iron-based superconductors, so the true predictive power of their model in these three families remains unexplored.

## 7. Spin- and Multi-Band–Mediated Pairing Mechanisms

Electron–differential phonon coupling, though insufficiently precise for *T_c_* predictions in IBSCs, might offer a viable route to improve the modeling. Here, we present spin-mediated mechanisms as plausible avenues to augment the approach in the future.

Across families such as 1111, 122, 11, and 111, iron-based superconductivity often emerges near antiferromagnetic (AFM) or spin-density-wave (SDW) phases and coexists with, or is proximate to, strong magnetic fluctuations. A widely supported scenario is that spin fluctuations, rather than lattice vibrations, act as the primary gluing mechanism for Cooper pairs. This section surveys the main ideas: the electronic structure that supports spin fluctuations, the mechanism by which these fluctuations couple to electrons, the resulting superconducting order parameter, the evidence supporting spin-fluctuation–mediated pairing, and the remaining puzzles.

IBSC shares a common multiband electronic structure dominated by Fe 3d states. The Fermi surface typically consists of hole pockets and electron pockets. Interband scattering between these pockets is strongly enhanced by the nesting conditions between hole and electron Fermi surfaces, often with a characteristic wave vector in the unfolded Brillouin zone. This nesting amplifies spin fluctuations at these wave vectors, producing a spectrum that is dominated by nearly AFM spin excitations. Crucially, the multiorbital character (d_xz_, d_yz_, d_xy_, and others) of iron orbitals means that spin fluctuations are not uniform across all orbitals. Orbital-selective correlations and interorbital scattering pathways give rise to a rich spin fluctuation landscape, including both itinerant (electron hole) and more localized magnetic tendencies [82]. The interplay between itinerant electrons and local moments, shaped by Hund’s coupling and orbital differentiation, sets the stage for a pairing mechanism that leverages spin dynamics rather than bare phonons. Orbital selectivity in the normal state leads to orbital-selective pairing in the superconducting state. This means electrons of a specific orbital character bind to form Cooper pairs, resulting in highly anisotropic superconducting energy gaps on the Fermi surface [82].

In a conventional phonon-mediated superconductor, lattice vibrations generate an effective attraction between electrons near the Fermi surface, leading to Cooper pairing with an isotropic or weakly anisotropic gap. In IBSC, the dominant idea is that virtual exchange of spin fluctuations provides the retarded, attractive interaction in a channel that favors sign-changing order parameters. Within a spin-fluctuation framework, the pairing interaction V(k, k) is mediated by the spin susceptibility Χ(q, ω). For electrons with momenta k and k′ on Fermi-surface sheets connected by q = k – k′, the exchange of a spin fluctuation with momentum q and energy ω contributes an interaction proportional to g2(χ(q, ω)), where g encodes the coupling between electrons and spin fluctuations. The key insight is that spin fluctuations favor a pairing state in which the superconducting gap changes sign between the nested Fermi surface sheets (e.g., between hole pockets at Γ and electron pockets at M). In the simplest two-band schematic, this leads to an s± pairing symmetry: the gap on hole pockets Δh is positive, while the gap on electron pockets Δe is negative (or vice versa). The sign change arises naturally from the repulsive interband interaction being converted into an attractive pairing channel when the order parameter has opposite signs on connected Fermi surfaces.

For multiorbital considerations, since IBSC involves multiple Fe d orbitals, the pairing interaction is not purely a function of momentum; it also depends on orbital character [82]. Spin fluctuations preferentially scatter electrons between orbitals that are strongly mixed in the nesting process. This orbital structure can enhance, or in some materials suppress, particular gap anisotropies and may produce complex gap textures, including accidental nodes or deep minima on certain Fermi-surface sheets. What the resonance tells us about the pairing glue is that the wave vector of the resonance mirrors the dominant spin fluctuations and the Fermi-surface topology that favors interband scattering between the nested pockets. In addition, impurities scatter electrons within the same band (intraband) and between bands (interband), where the s± state relies on a sign change between bands. Interband scattering acts similarly to magnetic scattering in conventional s-wave superconductors and can be pair-breaking. However, interband scattering can be incorporated into band-structure calculations, and multiorbital interactions can produce anisotropic Fermi surfaces, which may be partially incorporated into the model mentioned in Section 6.

Yet the rich material diversity of IBSC ensures that the full story remains nuanced, with ongoing exploration into orbital effects, nematicity, and the interplay between itinerant and local-moment physics. As research progresses, a more complete, material-specific map of how spin dynamics glue electrons into Cooper pairs may emerge, potentially guiding the design of new superconductors with higher *T_c_*.

## 8. Hope for the Future

As scientists continue to explore the fascinating realm of iron-based superconductors, the prospect of uncovering their pairing mechanism remains a tantalizing challenge. The strides made in enhancing the $T_{c}$ and the development of innovative theoretical models provide a solid foundation for future research endeavors. The mentioned experimental advancements in previous sections and sophisticated techniques are paving the way for deeper insights into the intricate interactions at play within these materials. Scientists are increasingly utilizing cutting-edge methods, such as high-resolution spectroscopy and advanced computational modeling, to reveal the underlying physics governing superconductivity. Moreover, collaborative efforts across disciplines, combining insights from materials science, condensed matter physics, and computational studies, hold the promise of accelerating our understanding of the pairing mechanisms. This multidisciplinary approach not only enriches our knowledge base but also inspires innovative solutions that could lead to new superconducting materials with even higher $T_{c}$ values. The future is bright as scientists forge ahead in this endeavor. The journey to uncover the pairing mechanism in iron-based superconductors is fraught with challenges, but it is also filled with immense potential. As scientists make new discoveries and refine models, they may find not only answers to the existing questions but also unforeseen opportunities for technological advancements in energy transmission, quantum computing, and other applications. With every experimental breakthrough and theoretical insight, scientists will draw closer to achieving a comprehensive understanding of these remarkable materials, inviting optimism for a future where the mysteries of iron-based superconductors are unveiled.

## Figures and Tables

**Table 2:** Relationship between the surface *xy*-strain and the $T_{c}$ in FeSe/STO, including the principal amplification factors responsible for strain-induced ${\;T}_{c}$ modulation. $R_{CDW}$ refers to the charge density wave factor, $R_{ARPES}$ refers to the unusual contribution from high-energy electrons as seen in ARPES experiment and $M_{Fe}$ denotes the magnetic moment on the Fe atom [42].

Compressive Strain on *x*- and *y*-Axis	$R_{ARPES}$	$R_{CDW}$	$M_{Fe}(\mu_{B})$	Calculated $T_{c}$
0%	1.30	2.9	1.29	98 K
1%	1.76	2.6	1.26	94 K
2%	1.52	2.3	0.71	86 K
2.5%	1.48	2.2	0.37	19 K
3%	1.47	2.2	0	0 K

**Table 3:** Experimental and theoretical superconducting *T_c_* in the selected IBSC [43,78].

	Experimental $T_{c}$ (K)	Theoretical $T_{c}$ (K)
BaFe_2_As_2_ (1.0 GPa)	~33	~35
BaFe_2_As_2_ (1.5 GPa)	~36	~37
BaFe_2_As_2_ (2.0 GPa)	~35	~35
BaFe_2_As_2_ (3.0 GPa)	~32	~33
BaFe_2_As_2_ (4.0 GPa)	~32	~34
BaFe_2_As_2_ (7.0 GPa)	~26	~28
Ba_0.8_K_0.2_Fe_2_As_2_	~26	~29
Ba_0.6_K_0.4_Fe_2_As_2_	~36	~32
Ba_0.4_K_0.6_Fe_2_As_2_	~27	~22
SrFe_2_As_2_ (3.0 GPa)	~30	~28
SrFe_2_As_2_ (5.0 GPa)	~19	~24
CaFe_2_As_2_ (0.1 GPa)	~13	~14
CaFe_2_As_2_ (1.2 GPa)	~13	~15
LiFeAs (2.5 GPa)	~15	~19
LiFeAs (4.5 GPa)	~12	~15
LiFeAs (6.0 GPa)	~10	~13
NaFeAs (0.0 GPa)	~11	~12
NaFeAs (1.0 GPa)	~10	~10
NaFeAs (2.0 GPa)	~13	~10
FeSe (0 GPa)	~10	~15
FeSe (1.0 GPa)	~15	~20
FeSe (2.0 GPa)	~20	~21
FeSe (3.0 GPa)	~22	~22
FeSe/STO	~100	~98

## Data Availability

No new data were created or analyzed in this study. Data sharing is not applicable to this article.
